# Tumor cell-induced platelet aggregation accelerates hematogenous metastasis of malignant melanoma by triggering macrophage recruitment

**DOI:** 10.1186/s13046-023-02856-1

**Published:** 2023-10-23

**Authors:** Yuyi Chen, Jie Zhou, Zishen Liu, Tongtong Wu, Shumeng Li, Yutong Zhang, Xiaohui Yin, Guowang Yang, Ganlin Zhang

**Affiliations:** 1 Department of Oncology, Shunyi Hospital, Beijing Hospital of Traditional Chinese Medicine, Beijing, China; 2 Department of Oncology, Beijing Hospital of Traditional Chinese Medicine, Capital Medical University, Beijing, China

**Keywords:** Tumor cell-induced platelet aggregation (TCIPA), Malignant melanoma (MM), Hematogenous metastasis, Tumor-associated macrophage (TAM)

## Abstract

**Background:**

Tumor cell-induced platelet aggregation (TCIPA) is not only a recognized mechanism for paraneoplastic thrombocytosis but also a potential breakthrough alternative for a low response to immune checkpoint inhibitors (ICIs) in hematogenous metastasis of malignant melanoma (MM). However, there is no TCIPA-specific model for further investigation of the relationship among TCIPA, the tumor immune microenvironment (TIME), and metastasis.

**Methods:**

We developed a TCIPA metastatic melanoma model with advanced hematogenous metastasis and enhanced TCIPA characteristics. We also investigated the pathway for TCIPA in the TIME.

**Results:**

We found that TCIPA triggers the recruitment of tumor-associated macrophages (TAMs) to lung metastases by secreting B16 cell-educated platelet-derived chemokines such as CCL2, SDF-1, and IL-1β. Larger quantities of TAMs in the TCIPA model were polarized to the M2 type by B16 cell reprocessing, and their surface programmed cell death 1 ligand 1 (PD-L1) expression was upregulated, ultimately assisting B16 cells in escaping host immunity and accelerating MM hematogenous metastasis.

**Conclusions:**

TCIPA accelerates MM lung metastasis via tumor-educated platelets (TEPs), triggering TAM recruitment, promoting TAM polarization (M2), and remodeling the suppressive TIME in lung metastases.

**Supplementary Information:**

The online version contains supplementary material available at 10.1186/s13046-023-02856-1.

## Background

Malignant melanoma (MM) is a grave challenge for global cancer control, with the mortality rate increasing to approximately 68% by 2040 [1]. The bloodstream spread and consequent pulmonary, cerebral, or other hematogenous metastases become the primary cause of MM death leading to a 4.6% 5-year survival rate [2]. Immune checkpoint inhibitors (ICIs) have been found to have a conclusive prognostic benefit for a variety of malignancies. However, over approximately half of metastatic MM cases with hematogenous metastasis respond poorly to ICIs [3]. Hunting for hidden targets causing immune escape acceleration has emerged as a key to breakthroughs in therapy for metastatic MM.

Paraneoplastic thrombocytosis [4] is a crucial phenomenon that occurs in patients with malignancies such as MM [5, 6] and presents as a series of symptoms, including enriched and aggregation-prone platelets [7, 8], elevated risks of developing secondary venous thromboembolisms (VTEs) and arterial thromboembolisms (ATEs) [9, 10], and even worse hematogenous metastasis.

Currently, the mechanism of paraneoplastic thrombocytosis is well recognized as tumor cell-induced platelet aggregation (TCIPA), the capacity of tumor cells to attract and induce platelets to cluster, release granules, and interact with the surrounding environment when the platelets and tumor cells contact or exchange biological information through adhesion molecules, cytokines, or extracellular vesicles [11]. Our team has recognized the integral role of TCIPA in the tumor metastasis cascade [12] for numerous growth and pro/anti-angiogenic factors, which are closely associated with tumor-specific abilities, such as proliferation, migration, invasion, adhesion, and angiogenesis, which has been reported in several published studies [13, 14]. Nevertheless, few studies have focused on the impact of TCIPA on the tumor immune microenvironment (TIME). This gap may be attributed to the lack of an accessible and stable TCIPA model that mimics paraneoplastic thrombocytosis, hindering the confirmation that TCIPA acts on the host in vivo.

In this study, we established a TCIPA metastatic melanoma model to investigate TCIPA in the melanoma microenvironment and its role in hematogenous metastasis. Our findings illustrate that TCIPA accelerates melanoma lung metastasis without directly promoting tumor cell migration. Tumor cell-educated platelets (TEPs) trigger the recruitment of tumor-associated macrophages (TAMs), participate in inducing TAM polarization, and reshape the suppressive TIME in lung metastases.

## Materials and methods

### Cells and mice

The mouse melanoma cell line B16-F10-Luc-G5 (B16 cells) and the mouse Lewis lung cancer cell line LL/2-Luc-M38 (LL/2 cells) were purchased from Shanghai Genomics Technology, Ltd. FaDu human pharyngeal squamous carcinoma cells were kindly donated by Prof. Israel Vlodavsky (Technion Integrated Cancer Center, Rappaport Faculty of Medicine). The human ovarian cancer cell line A2780 was purchased from Hunan Fenghui Biological Ltd. The mouse mononuclear macrophage leukemia cell line RAW 264.7 was obtained from the National Infrastructure of Cell Line Resource (NICR, Beijing, China). B16, A2780 and RAW 264.7 cells were incubated in RPMI1640 (Gibco, Invitrogen, Grand Island, USA) with 10% FBS (Gibco, Invitrogen), 100 U/mL penicillin, and 100 mg/mL streptomycin at 37 °C in 5% CO_2_, while LL/2 and FaDu cells were cultured in DMEM under the same conditions.

C57BL/6N mice were purchased from Beijing Vital River Laboratory Animal Technology Co. Ltd. Six- to eight-week-old male mice were used for the in vivo assay, while those aged 18–20 weeks were used for washed platelet preparation. This study was approved by the Animal Ethics Committee of the Laboratory Animal Center, Beijing Hospital of Traditional Chinese Medicine (No.2019120202).

### Washed platelet preparation and calculation

Washed platelets were isolated from fresh whole blood from age-matched mice or venous blood from healthy volunteers at room temperature. Typically, blood was collected in 3.2% sodium citrate-treated tubes and gently mixed. The samples were spun at 156 × g for 7 min in an Allegra X30R centrifuge (Beckman, USA) with no brake. Following that, the blood constituents were observed to be split into three layers: the top layer of straw-colored platelet-rich plasma (PRP), the middle ‘buffy coat’, and the bottom red blood cell (RBC) layer. PRP was transferred into new Eppendorf tubes for a second centrifugation (300 × g, 4 min, with no brake) to remove any residual white blood cells (WBCs) and RBCs. Pure PRP was then spun at 1000 × g for 4 min with no brake. After discarding the supernatant, washed platelets were resuspended in buffer (137 mM NaCl, 12 mM NaHCO_3_, 2.6 mM KCl).

The platelet suspension was diluted in an aqueous solution (10 mg/mL ammonium oxalate, 0.12 mg/mL EDTA·Na_2_, and 0.8% paraformaldehyde(PFA)). The number of nonactivated washed platelets was calculated using a hemocytometer, and the suspension was adjusted to the proper concentration.

### Establishment of TCIPA metastatic melanoma model (B16 + PLT model)

B16 cells and platelets were adjusted to 1 × 10^7^/mL and 1 × 10^9^/mL, respectively. The metastatic melanoma model (B16 model) was established by injecting 100 μL of B16 cell suspension through the tail vein, while 40 μL of the suspension was subcutaneously injected into the groin. We injected 100 μL of platelets through the tail vein before injection of B16 cells, for adequate contact and entanglement in the circulation to build the TCIPA metastatic melanoma model (B16 + PLT model). In the B16 + PLT model, the final concentration ratio of injected platelets to B16 cells was 100:1. Follow-up experiments revealed that the B16 + PLT model produced the expected enhanced TCIPA phenomenon in vivo.

Body weight and primary tumor size were recorded during observation. On the 14^th^ day, all the mice were sacrificed. Peripheral platelets were counted using an Automatic Five-Category Blood Cell Analyzer (Sysmex, XT-2000i, Japan). Lung tissues and primary tumors were photographed and preserved in 4% PFA for hematoxylin and eosin (H&E) staining.

To test our hypothesis, 100 μL of InVivoMab anti-mouse programmed death ligand 1 (PD-L1) antibody (1 mg/mL, Bio X Cell, catalog no.BE0101, USA) was injected intraperitoneally on days 3, 6, 9, and 12 after model establishment. The observation methods and indicators have been described above.

### B16 and other tumor cells induced platelet aggregation in vitro

B16 and LL/2 cells were used for TCIPA assays in vitro. Interactions between tumor cells and platelets in vitro were observed using a light transmission aggregometry (LTA) assay. First, 250 μL of 1 × 10^9^/mL washed platelets was incubated at 37 °C for 5 min with magnetic rod stirring at 900 rpm. TCIPA was initiated by adding 1 × 10^7^/mL B16 or LL/2 cells in 50 μL. The reaction was monitored for 10 min and analyzed using an LBY-NJ semiautomatic platelet aggregometer (PrismLab, Beijing, China). When detecting FaDu cell-induced platelet aggregation, 250 μL of PRP was used in place of washed platelets, as before.

### Immunohistochemistry (IHC) assay

Lung tissue was harvested from B16 + PLT model, B16 model, and control mice, with three samples in each group. After antigen retrieval, paraffin slides were incubated with 3% BSA to block nonspecific binding.

For platelet infiltration and activation, an anti-CD42b rabbit polyclonal antibody (Affinity, catalog no.DF8519) and anti-CD62p rabbit polyclonal antibody (Affinity, catalog no.DF13294) with the optimal dilution of 1:100 were incubated with slides at 37 °C for 60 min, respectively. Then, biotin-labeled anti-rabbit goat IgG and a horseradish enzyme-labeled streptavidin solution were added successively before the chromogenic agent DAB, and the cells were washed three times with PBS at intervals. The nuclei were stained with hematoxylin. For macrophage infiltration, an anti-F4/80 rabbit polyclonal antibody (Affinity, catalog no.DF2789, 1:180) and anti-MRC1 rabbit polyclonal antibody (Affinity, catalog no.DFDF4149, 1:50) were used. PD-L1/CD274 rabbit polyclonal antibody (Proteintech, catalog no.17952–1-AP, 1:600), anti-C–C motif chemokine receptor 2 (CCR2) antibody (Abcam, catalog no. ab273050, 1:250), anti-interleukin-1 receptor 1 (IL1R1) rabbit polyclonal antibody (Sino Biological, catalog no. 50807-T24, 1:1000), anti-C-X-C motif chemokine receptor 4 (CXCR4) antibody (Abcam, catalog no. ab181020, 1:500) were used to observe the expression of the corresponding proteins. Five fields were selected for each stained slide and measured using Image-Pro Plus (version 6.0.0.260) software.

### Modified MSB staining assay

For assessing the TCIPA complex in vivo, a modified Martius Scarlet Blue (MSB) (Solarbio, catalog no.G2040, Beijing, China) method [15] was used (Supplementary Material 2). The pathological features of the lung tissues were identified by microscopy.

### Transwell migration assay for recruitment

RAW 264.7 cells were seeded in the upper compartment of a 8 μm Transwell insert at a concentration of 5 × 10^5^/mL with 100 μL of FBS-free RPMI 1640. The compartments contained control culture medium (RPMI 1640/DMEM), self-activated platelets (platelets only), tumor cells (tumor cells only), or a TCIPA mixture (tumor cell-to-platelet ratio was 1:100). These systems, which were cocultured for 12 h in advance, were centrifuged at 3200 × g for 10 min and filtered through a 0.22 μm membrane for later use. Then, 700 μL of the supernatant from each group was added to the lower compartment, with FBS adjusted to 20%.

### Flow cytometry

Fresh spleen, lung, and tumor tissues were removed from the mice immediately after sacrifice and prepared as single-cell suspensions at 5 × 10^6^/mL. Antibody labeling was performed using Fc-receptor blockers [Purified Rat Anti-Mouse CD16/CD32 antibody (BD, catalog no. 553141, USA), True-Stain Monocyte Blocker (BioLegend, catalog no. 426102, USA)], and Zombie NIR Fixable Viability Kit (BioLegend, catalog no. 423105, USA). The RedFluor™ 710 Anti-Mouse CD45 (30-F-11) (TONBO Biosciences, catalog no. 80–0451, USA), PerCP-Cyanine5.5 anti-Mouse F4/80 antigen (BM8.1) (TONBO Biosciences, catalog no. 65–4801, USA), CD11b monoclonal antibody (M1/70) eFluor 450 (eBioscience, catalog no. 48–0112-82, USA), CD206 (MMR) monoclonal antibody (MR6F3) PE-Cyanine7 (eBioscience, catalog no. 25–2061-82, USA) were applied for staining according to the manufacturer's instructions. In vitro, CD86 BV421 (Biolegend, 105031, GL-1) and anti-PDL1 PE (BD, 558091, MIH5) antibodies were added to the staining scheme. The stained single-cell suspensions were assessed by a BD LSR Fortessa flow cytometer (BD Biosciences, USA) with FlowJo (version 10) software.

### ELISA

An ELISA was used to detect the release of TCIPA. The test samples were collected from the serum and lung tissue homogenate supernatants of three mice in each group. Mouse c–c motif chemokine ligand 2/monocyte chemotactic protein-1 (CCL2/MCP-1, catalog no. EK0568), mouse interleukin-1 alpha (IL-1α, catalog no. EK0391), mouse interleukin-1 beta (IL-1β, catalog no. EK0394), mouse macrophage-stimulating factor (M-CSF, catalog no. EK0445), mouse transforming growth factor-beta 1 (TGFβ1, catalog no. EK0515), mouse interferon-gamma (IFN-γ, catalog no. EK0375), mouse interleukin-4 (IL-4, catalog no. EK0405) and interleukin-10 (IL-10, catalog no. EK0417) ELISA kits were purchased from Boster Biological Technology Co., Ltd. (Wuhan, China). Mouse macrophage migration inhibitory factor (MIF, catalog no. H598-M-96, Nanjing, China) and mouse stromal cell-derived factor 1 (SDF-1, catalog no. H398-1-M-96, Nanjing, China) were purchased from Nanjing Jiancheng Bioengineering Institute.

### Wound healing assay

A 200 μL sterile micropipette tip was used to make a straight-edged wound in each well in which 5 × 10^6^/mL tumor cells were precultured for 8 h. After washing away cell debris with PBS, platelets at a ratio of 100:1 tumor cell were added at hour 0 of the coculture. Three images were captured at 0 h and the endpoint per well, which was the following for the different cells: B16 cells at 18 h, LL/2 cells at 24 h, FaDu cells at 22 h; there were three wells for each group. The observation time was determined according to the fastest healing group. Changes in the wound areas were calculated using ImageJ (version 1.52a) software.

### MTT assay

B16 cells and washed platelets were resuspended in FBS-free RPMI 1640 in proportions of 1:50, 1:100, 1:200, 1:400, and 1:800, and four groups (RPMI 1640, PRMI 1640 + platelet wash buffer, self-activated platelets, and B16 group) were set up as controls. The interaction system was then placed in a thermostatic culture oscillator at 37 °C, 60 rpm for 2 h. After centrifugation at 3200 × g for 10 min, the supernatant was collected and filtered. Considering that the B16 cell-platelet interaction consumes FBS, we adjusted the FBS concentration to 10% before adding the medium to 96-well plates coated with B16 cells (1.5 × 10^4^/mL, 8 h in advance). MTT (Sigma‒Aldrich, catalog no. M5655, USA) was used for absorbance measurements.

### Statistical analysis

IBM-SPSS (version 25.0) was used to perform statistical analyses. The statistical significance threshold was set at *p* ≤ 0.05. One-way ANOVA and LSD tests were used for data with uniform variances. Mann–Whitney U test was used for data that didn't fit a normal distribution. An unpaired (two-tailed) t test was used to compare two groups using ELISA. Error bars represent the SEM. *p ≤ 0.05, **p ≤ 0.01, ***p ≤ 0.001.

## Results

### The B16 cell:PLT ratio is a crucial determinant of the characteristics of the metastatic melanoma model

**Fig. 1:**
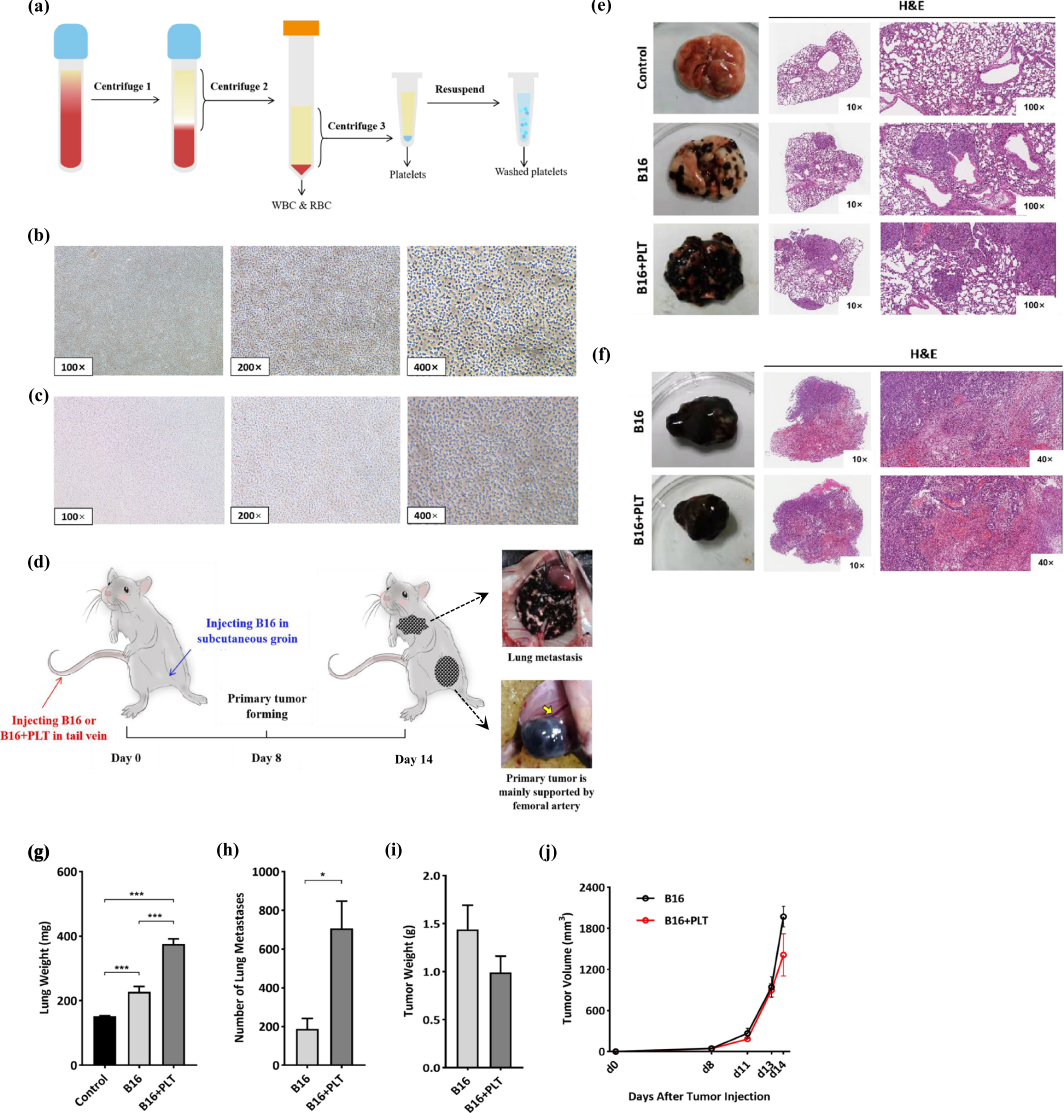
Preparation of washed platelets and establishment of the TCIPA metastatic melanoma model (B16 + PLT model). **a** Scheme for the preparation of washed platelets. WBC, white blood cell; RBC, red blood cell; centrifuge 1: 156 × g for 7 min, centrifuge 2: 300 × g for 7 min, centrifuge 3: 1000 × g for 4 min, all with no brake. **b** The human-derived and (**c**) mouse-derived washed platelets were observed under 100 × , 200 × , and 400 × magnification. **d** Flowchart for establishment of the metastatic melanoma model (B16 model) and the TCIPA metastatic melanoma model (B16 + PLT model). Photographs and H&E staining images of (**e**) lung metastases and (**f**) primary tumors in the B16 + PLT model, B16 model and control groups. **g**-**h** When compared with the B16 model, the B16 + PLT model produced a significantly greater number of lung metastases (*N* = 8) and heavier lung weight (*N* = 9). **i**-**j** For the primary tumor weight and volumes (*N* = 6), there was no significant difference between the two models. *p ≤ 0.05, ***p ≤ 0.001

To determine the role of platelets in hematogenous metastasis of melanoma, we first optimized the reported protocol [16–20] for platelet isolation, as shown in Fig. 1a. The washed platelets were elliptical discs of 2–3 μm with smooth membranes that refracted light in blue color and separated from each other (Fig. 1b-c). This observation implies that our washed platelets remained in a resting state without aggregation or activation, which greatly avoids the risk of injecting emboli into the vessel and causing pulmonary embolism.

We then built a pair of models: the metastatic melanoma model (B16 model) and the corresponding TCIPA model (B16 + PLT model), using the scheme shown in Fig. 1d. These two simulated advanced melanoma patients with or without thrombocythemia. Previous clinical trials [21, 22] have demonstrated that an increase in circulating platelet levels can accelerate tumor metastasis. However, no study has explored the specific extent of platelet increase. We initially set the ratios of B16 cells to platelets to 1:50, 1:100, and 1:200. As shown in Figure S1, lung weights increased in the 1:50 (*p* = 0.007) and 1:100 (*p* = 0.019) groups compared to those in the B16 model, but the difference was not significant at 1:200. Therefore, the ratio must be within a specific range, 1:50 to 1:100 in our study, for an increase in platelet levels to promotes lung metastasis in melanoma. Finally, a ratio of 1:100 was selected to complete subsequent experiments.

### Advanced hematogenous metastasis in the TCIPA metastatic melanoma model

Repeating the construction of the TCIPA model (B16 cell:PLT = 1:100), we found a significant increase again in lung weight versus that in the B16 model and control (375.56 ± 47.99 mg vs. 222.78 ± 50.19 mg vs. 152.22 ± 4.41 mg, *p* ≤ 0.001). Moreover, the B16 + PLT model exhibited more metastases in the lung, kidney, liver, and colon (Fig. 1h and Table 1). This demonstrates that platelets accelerate the hematogenous metastasis of melanoma and not merely lung metastasis, which is the most obvious manifestation. However, the primary tumors tended to nonsignificantly shrink in the TCIPA model, which is in accordance with a published study [23]. Thus, we affirmed that the TCIPA model has more advanced hematogenous metastasis.

**Table 1:** Other organs metastasis [Median (P25, P75)]

Organs	B16 model	B16 + PLT model
Kidney	0.50 (0.00, 1.75)	5.00 (2.00, 7.00)
Liver	0.00 (0.00, 0.00)	4.00 (0.75, 6.50)
Colon	0.00 (0.00, 0.75)	3.00 (3.00, 3.50)

Mann–Whitney U test, *p* = 0.10

### Enhanced TCIPA characteristics in vivo were found in the TCIPA metastatic melanoma model

**Fig. 2:**
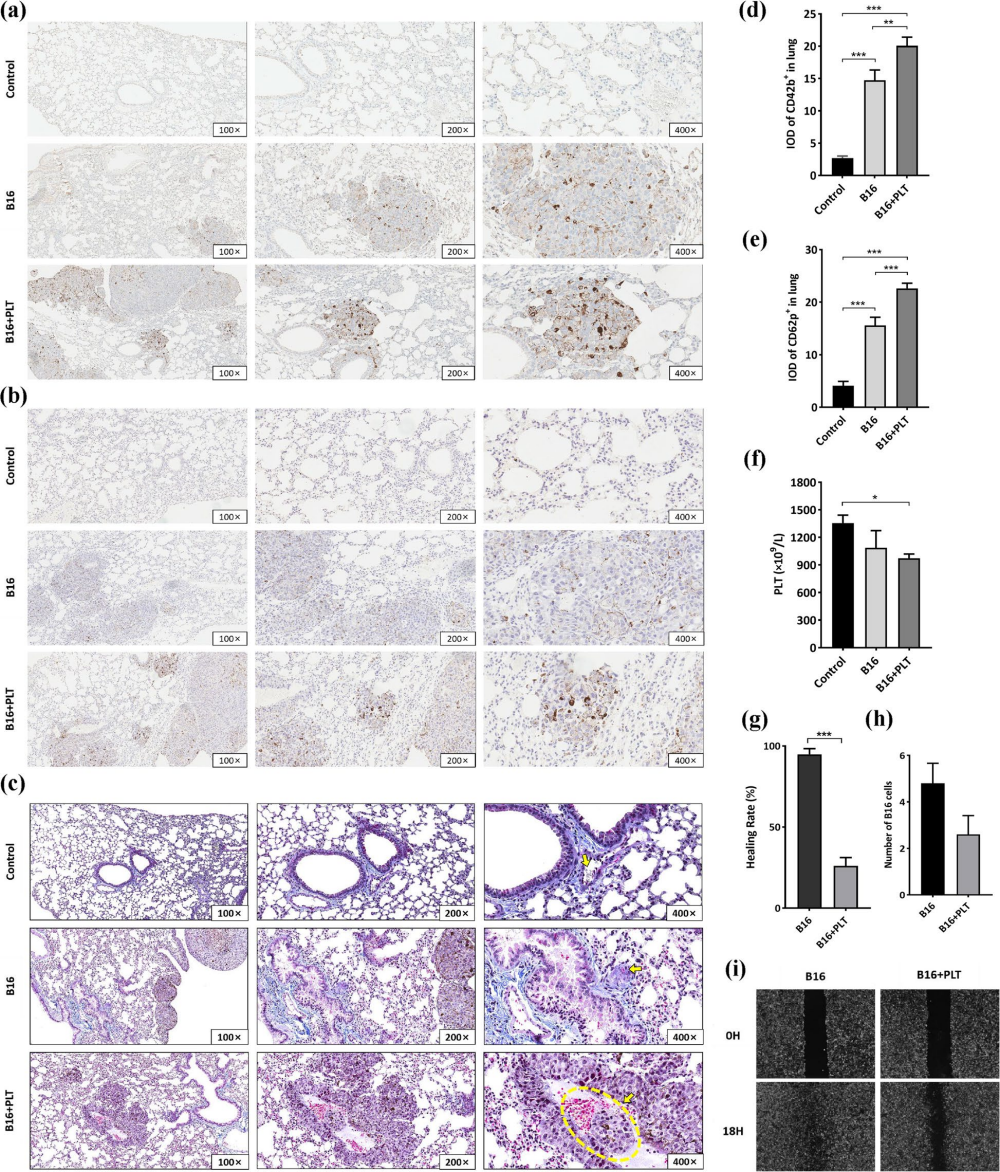
TCIPA in vivo is enhanced in the B16 + PLT model but does not directly promote the migratory capacity of B16 cells. The expression of (**a**) CD42b and (**b**) CD62p in the lung metastases was observed by IHC assay under 100 × , 200 × , and 400 × magnification. Chocolate brown: positive expression; calamine blue: nucleus. As shown in graphs (**d**) and (**e**), the integrated optical density (IOD) values of CD42b and CD62p were significantly different between the B16 + PLT and B16 models. **p ≤ 0.01, ***p ≤ 0.001, *N* = 15. **c** Modified MSB staining images. Incarnadine: fresh fibrin; bluish violet: stale fibrin; bluish-brown: nucleus; yellow: RBCs; sapphire: collagen fibers; gray: platelet trabeculae. **f** Peripheral platelet count. *p ≤ 0.05, *N* = 5. **g**-**i** Platelet-stimulated B16 cells showed neither faster wound healing (***p ≤ 0.001, *N* = 3) nor greater migratory capacity than B16 cells alone (no significant difference, *N* = 5)

We were curious whether the TCIPA model resulted in a stronger TCIPA response in vivo? To address this question, we first confirmed that tumor cells such as B16, LL/2, and FaDu cells were all capable of inducing platelet aggregation in vitro (Figure S2). Therefore, we investigated where the B16 cells transported the injected platelets. Through peripheral platelet counting and IHC, we found that the extra platelets did not stay in circulation but rather infiltrated the lung metastasis tissues (Fig. 2a and 2d). The infiltration (based on tracking CD42b, a platelet-specific membrane glycoprotein, GPIbα) and activation (CD62p, a glycoprotein that indicates α granule release and platelet activation) of infiltrating platelets in the TCIPA model were much greater than those in the B16 model and the control (Fig. 2b and 2e). However, we were still unable to observe the TCIPA process in vivo via these assaies.

TCIPA leads to mutual stimulation, aggregation, and membrane fusion between tumor cells and platelets [23]. These tumor-cell-educated platelets release procoagulant substances and participate in thrombosis, which was described by Fidler IJ in "Embolism: interaction with platelets, lymphocytes and other blood components" [24]. Accordingly, we attempted to identify tumor cells, fibrin, interior activated and exterior resting platelets [25], and multiple blood cells trapped by platelet trabeculae and fibrin meshwork in thrombi via modified MSB staining. As shown in Fig. 2c, the arrow in the control image (400 ×) indicates that gray disc-shaped cells deposited within the vasculature, with a diameter half that of RBCs, are considered platelets with intact membranes in the resting state. In the B16 model (400 ×), fresh fibrin was found in lung metastases in addition to the bronchi, suggesting the existence of microthrombus in metastatic foci. The arrow in the image from the TCIPA model (400 ×) shows a complex consisting of B16 cell masses, erythrocytes, scattered inflammatory cells, intertwined fresh fibrin, and platelet trabeculae, blocking in the vascular lumen, which most probably represents a TCIPA complex in vivo.

Theses phenomena together indicate that enhanced TCIPA occurred in our TCIPA model.

### Platelets cannot directly promote the migratory capacity of B16 cells in vitro

According to previous studies by our team [13, 26], platelet-derived release can directly impel tumor cells to become more aggressive. Therefore, we speculated that B16 cell-educated platelets accelerate melanoma hematogenous metastasis by promoting B16 cell migration. However, the wound-healing rate of B16 cells was not improved after coculturing with platelets in vitro compared with that of B16 cells alone (Fig. 2g and i). We retested migration using the Transwell method and obtained a similar result (Fig. 2h). We also showed that the negative result was not related to the effect of platelets on B16 cell proliferation (Figure S3). Indeed, by expanding to the FaDu, LL/2, and A2780 cells, the impact of platelets was found to be distinct across cancer species (Figure S4).

According to these results, TCIPA probably does not worsen melanoma metastasis by enhancing the migratory capacity of B16 cells. Since the direct mechanism was excluded, we wondered whether TCIPA could lead to lung metastasis aggravation via an indirect mechanism, such as altering other host characteristics.

### TCIPA reshapes the host TIME, especially the characteristics of macrophages in lung metastases

**Fig. 3:**
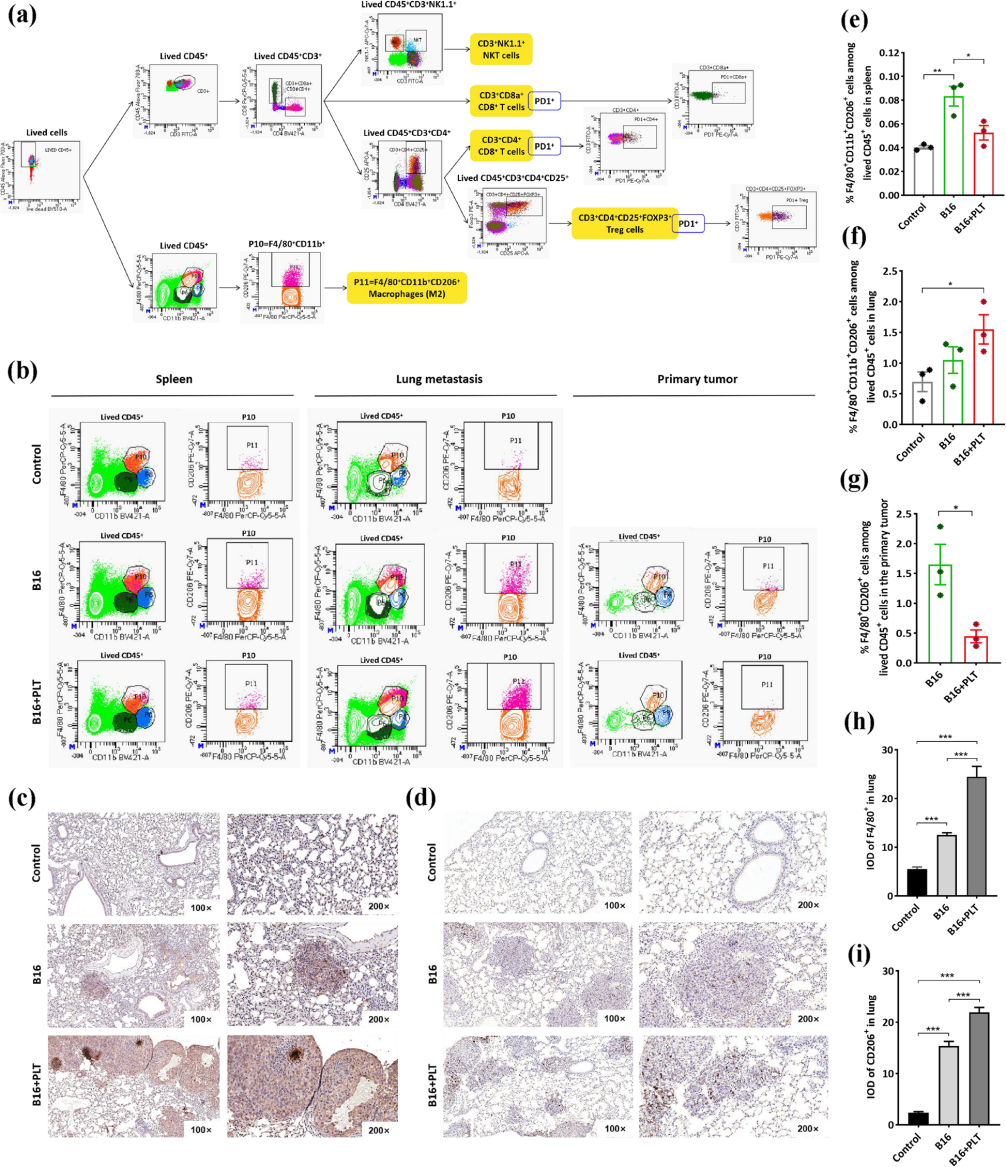
TCIPA alters the phenotype and number of macrophages in the TIME. **a** The FCM protocol. **b** The proportion of F4/80^+^ CD11b^+^ CD206^+^ cells among the live CD45^+^ cells in the fresh spleens (**e**), lung metastases (**f**) and primary tumors (**g**) from the B16 + PLT model, B16 model and control groups. *p ≤ 0.05, **p ≤ 0.01, *N* = 3. The expression of F4/80 (**c**) and CD206 (**d**) in lung metastases was detected by IHC. The IOD values of both F4/80 (**h**) and CD206 (**i**) were significantly higher in the B16 + PLT model than in the B16 model. ***p ≤ 0.001, *N* = 15

Based on the spleen and thymus indexes (Figure S5), we speculated that TCIPA might alter the adjustment of host immunity. To determine the immune cell populations that were affected, we randomly selected fresh spleens, lungs, and primary tumors from the TCIPA model, B16 model, and control mice and performed FCM according to the protocol shown in Fig. 3a.

We found that platelets, as “first responders” in the immune regulation pathway [27], have a wide range of effects on both host immunity and the TIME. The most dramatic difference was observed in macrophages (Fig. 3b). The proportion of the F4/80^+^CD11b^+^CD206^+^ population suggestive of the M2 phenotype was significantly increased in the lung but decreased in the primary tumor and spleen of TCIPA model mice (Fig. 3e-g). In addition, programmed cell death protein 1 (PD-1) expression was obviously upregulated in NKT and Treg cells in TCIPA model lungs, as well as in NK cells in situ (Figure S6b-d). Small fluctuations were also observed between CD4^+^ and CD8^+^ T cells in the spleen (Figure S6a). These FCM results suggested that TCIPA most likely shifted the TIME toward immunosuppression in lung metastases. Then, we confirmed this hypothesis by IHC. Here, we found that the infiltration of F4/80^+^ and CD206^+^ cells in the TCIPA model was not only increased (Fig. 3h-i) but also occurred within the lung metastatic lesions (Fig. 3c-d).

The macrophages in the TIME can be distinguished as macrophages in the local stroma and tumor-associated macrophages (TAMs) [28]. Thus, we sought to determine which types of macrophages are specifically enriched in lung metastases by TCIPA.

### TCIPA induced TAM recruitment in lung metastases

**Fig. 4:**
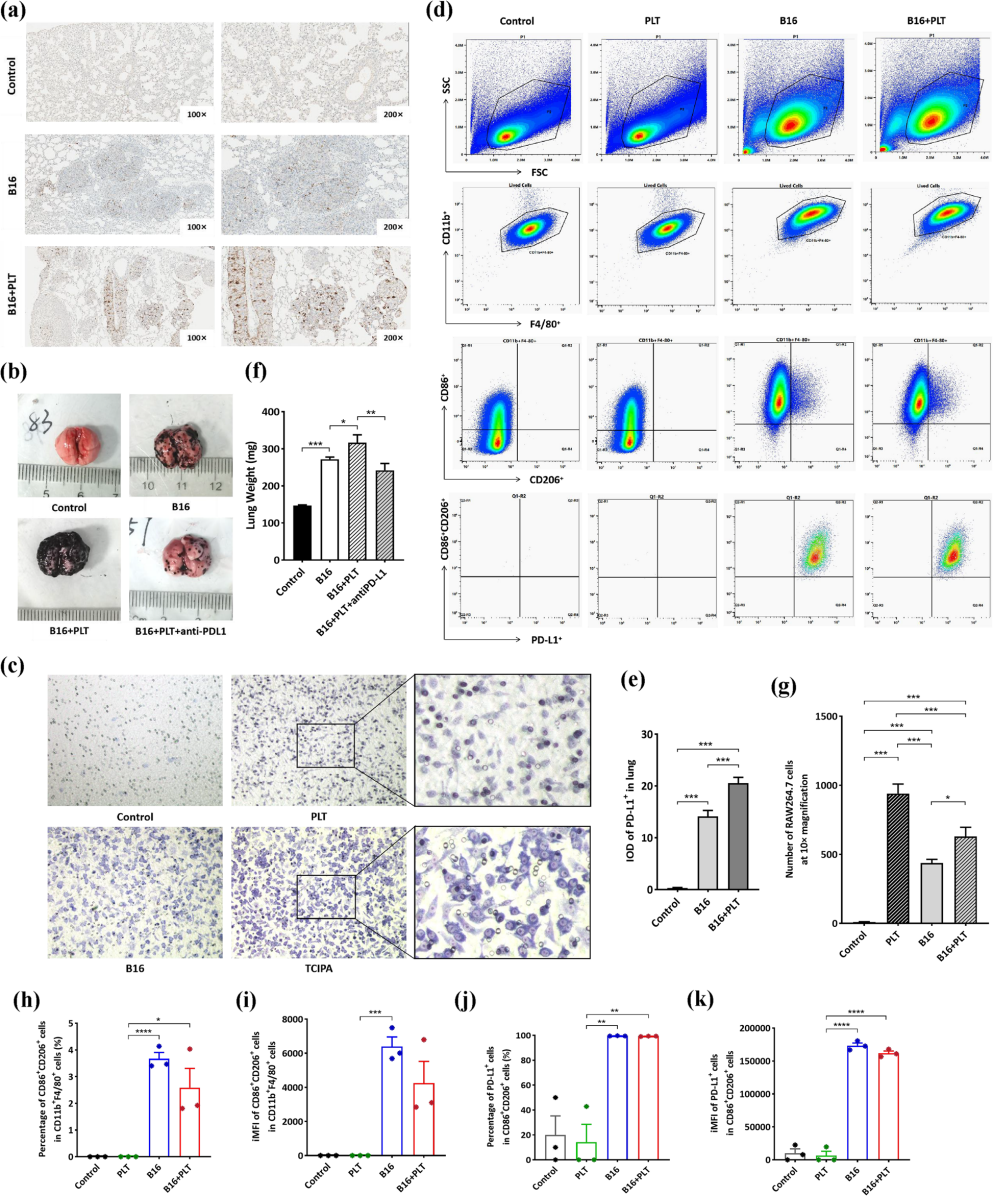
TCIPA promotes macrophage recruitment to lung metastases, stimulates TAM to M2 phenotype, and upregulates its PD-L1 expression. **a** and **e** The expression of PD-L1 in lung metastases. ***p ≤ 0.001, *N* = 15. **b** and **f** Changes in lung weight after InVivoMab anti-mouse PD-L1 treatment. **p ≤ 0.01, *N* = 6. **c** and **g** Migratory capacity of RAW 264.7 cells under the attraction of RPMI 1640, PLT, B16, and PLT + B16 treatments as determined by Transwell assay. *p ≤ 0.05, ***p ≤ 0.001, *N* = 5. **d** RAW264.7 cells were grouped as described in (**c**). FSC + SSC suggested the granularity of the live RAW264.7 cells. The macrophage gate was defined by CD11b^+^ and F4/80^+^. CD86^+^ and CD206^+^ gating coselected macrophages with the M2 phenotype. Sorting was conducted for PD-L1 expression on the surface of macrophages (M2). **h**–**k** The proportion and integrated mean fluorescence intensity (iMFI) of CD86^+^CD206^+^ cells among CD11b^+^F4/80^+^ cells and PD-L1^+^ cells among CD86^+^CD206.^+^ cells. *p ≤ 0.05, **p ≤ 0.01, ****p ≤ 0.0001, *N* = 3

TAMs evolve from monocytes recruited to tumor tissue. The capacity of TCIPA to induce monocyte recruitment is essential in identifying whether the enriched macrophages are TAMs. As shown in Fig. 4c, more RAW 264.7 cells crossed the polycarbonate membrane in the B16 + PLT group than in the B16 group (629.00 ± 67.59 vs. 436.20 ± 27.91,* p* < 0.001) under the attraction of RPMI 1640, PLT, B16 and PLT + B16 supernatants (containing 20% FBS). Thus, TCIPA promotes macrophage recruitment. This suggests that a substantial portion of the enriched macrophages in the TCIPA model lung are TAMs.

Surprisingly, spontaneously activated platelets recruited the greatest number of RAW264.7 cells independently (Fig. 4g). However, the RAW 264.7 cells recruited by platelets alone were shuttle-shaped with short protrusions on the surface. In contrast, those in the B16 + PLT group were irregularly shaped, with long pseudopods spreading and small dark nuclei.This phenomenon suggests that RAW 264.7 cells were activated by B16 cell-educated platelets, whereas platelets fail to do so themselves. We next performed an FCM assay on each group of RAW264.7 cells. The SSC values of TCIPA-recruited RAW264.7 cells, representing cell granularity, were found to be much larger than those of platelet-recruited cells (Fig. 4d). These features implied that TCIPA-recruited RAW264.7 cells were probably activated.

### TCIPA affects TAM polarization and PD-L1 expression on the TAM surface

Furthermore, we found that TAM polarization to M2-type TAMs was induced by TCIPA treatment and B16 cells, and there was no significant difference (Fig. 4h and i). However, a significantly higher percentage of CD86 ^+^ CD206 ^+^ TAMs was recruited by TCIPA treatment than by platelets. These results indicate that the increased number of M2-type TAMs in TCIPA-induced lung metastases is attributable to the recruitment of more TAMs by B16 cell-educated platelets.

According to previous studies [29, 30], increased PD-L1 levels on TAMs inhibits the recognition and defense functions of cytotoxic cells, ultimately leading to the immune escape of tumor cells. Therefore, we investigated whether TCIPA would further impact PD-L1 expression on the surface of M2-type TAMs. First, we found by IHC that total PD-L1 expression was significantly enhanced in TCIPA-induced lung metastases compared to that in the B16 model and control (Fig. 4a and e), tentatively supporting this hypothesis.Next, the TCIPA model mice were was treated with InVivoMab anti-mouse PD-L1 antibody. The lung weight of mice in the B16 + PLT group were reduced by 23.68% after PD-L1 blockade (Fig. 4b and f). Finally, the PD-L1-positive rate was almost 100% in both TCIPA treatment- and B16 cell-polarized TAMs as determined by FCM (Fig. 4j and k). These results indicate that B16 cells have a monopolistic effect in regulating PD-L1 expression on M2-type TAMs in the TCIPA process, whereas B16 cell-educated platelets tend to primarily affect TAM recruitment.

**Fig. 5:**
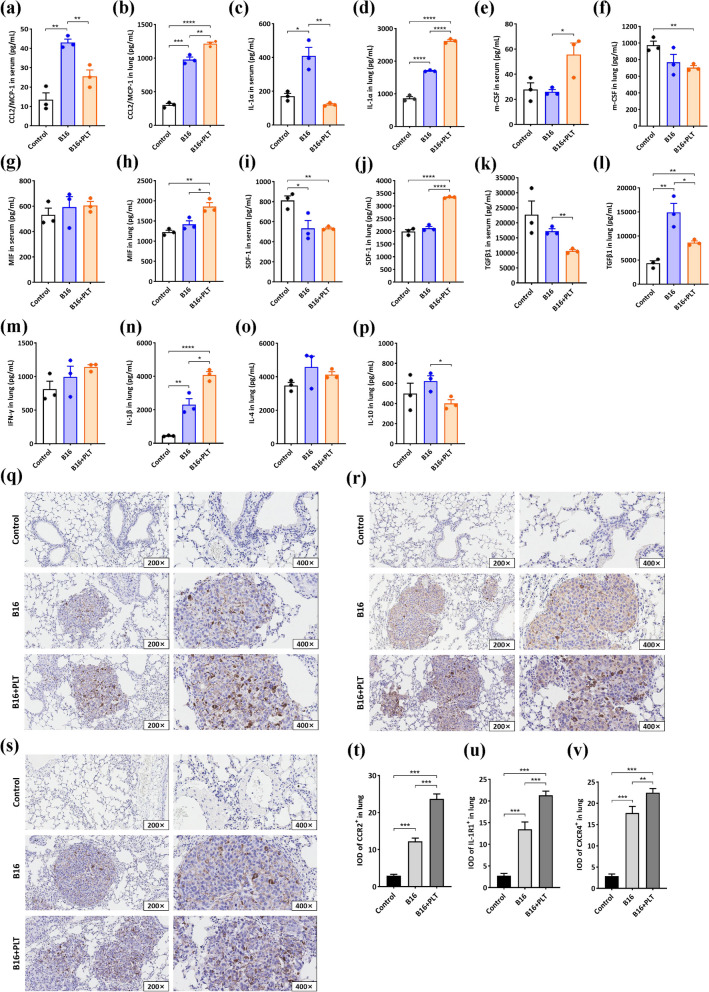
TCIPA-induced cytokine release triggers macrophage recruitment. Chemokines and growth factors in the serum or lung of the B16 + PLT model, B16 model and control groups, including CCL2/MCP-1 (**a**-**b**), IL-1α (**c**-**d**), m-CSF (**e**–**f**), MIF (**g**-**h**), SDF-1 (**i**-**j**), TGFβ-1 (**k**-**l**), IFN-γ (**m**), IL-1β (**n**), IL-4 (**o**), IL-10 (**p**) (pg/mL). *p ≤ 0.05, **p ≤ 0.01, ***p ≤ 0.001, ****p ≤ 0.0001, *N* = 3. The expression of CCR2 (q), IL-1R1(r) and CXCR4 (s) in lung metastases was detected by IHC. Their IOD values (t-v) were all significantly higher in the B16 + PLT model than in the B16 model. **p ≤ 0.01, ***p ≤ 0.001, *N* = 15

### TCIPA triggers TAM recruitment via chemokine release

To clarify the mechanism by which TCIPA stimulates TAM recruitment, we tested several cytokines in the lung suspensions and/or serum of the TCIPA model, B16 model, and control in vivo (Fig. 5a-p). CCL2/MCP-1, SDF-1, IL-1α, and IL-1β levels were significantly elevated in the context of TCIPA-induced lung metastases. Among them, CCL2 and SDF-1 are major determinants of macrophage recruitment and localization [31, 32]. Additionally, TGF-β, IL-10, and m-CSF levels were notably reduced in the lungs, but the concentration of m-CSF concentration in the serum was increased. Moreover, the expression of CCR2 (the receptor for CCL2), IL-1R1 (one of the major receptors for IL-1α and IL-1β) and CXCR4 (the receptor for SDF-1) was enhanced in the context of TCIPA-induced lung metastasis (Fig. 5q-v). This result at least suggests that TCIPA triggers TAM recruitment via the release of CCL2, SDF-1, IL-1α and IL-1β, which activate their receptors CCR2, IL-1R1 and CXCR4, respectively, and stimulates peripheral monocytes to migrate into tissues and differentiate into TAMs [33].

## Discussion

The role of TCIPA or tumor cell-educated platelets in tumor metastasis has been controversial [34, 35]. To explore therapeutic strategies for advanced melanoma, our study provides insight into the TCIPA mechanism based on a novel TCIPA metastatic melanoma model.

Prior to this study, previous research seek platelet-associated biomarkes was based on a tumor-bearing model alone [14, 36]. Such models can offer preliminary evidence of TCIPA-related pathways but fail to separate platelets as an independent variable. Papa AL and his colleagues [37] built a metastatic model by coincubating MDA-MB-231 cells with washed platelets at a ratio of 1:438 for 30 min and intracardially injecting the pretreated cells into mice. However, our team demonstrated a significant difference in the proliferation, migration, and invasion of MDA-MB-231 cells between 30 min and 24 h of pretreatment [13]. Therefore, Papa’s model was more appropriate than previous approaches for studying TCIPA in vivo, except the time dependence of TCIPA was not addressed. We also considered a tumor-bearing model treated with recombinant thrombopoietin that might simulate paraneoplastic thrombocytosis upon abnormal secretion in various malignancies [38, 39]. However, the adverse effects of such platelet regulators become inevitable confounders, including myelofibrosis, hematologic malignancies, rebound thrombocytopenia caused by drugs, and the effect of these reactions on the TIME. Ultimately, we established a TCIPA metastatic melanoma model (B16 + PLT model) to observe secondary hematogenous metastasis under the burden of primary melanoma induced by TCIPA.

Almost all clinical trials have shown that thrombocytosis is an independent risk factor for aggravated pathogenesis and poor prognosis in malignancies [40, 41]. Consistently, our TCIPA model demonstrated that an increase in platelet levels in a specific range leads to aggravation of hematogenous metastases. This range depends on the number of circulating tumor cells (CTCs), and we evaluated a B16 cell:PLT ratio of 1:50–200 in our study.

However, conclusive evidence for TCIPA remains to be obtained. LTA is currently the standard assay for assessing platelet aggregation in vitro [42]. In addition to LTA, all-electron microscopy [43], FCM [44], IHC [45] and in vivo multiphoton laser scanning microscopy [46] are recognized methods for TCIPA observation. However, evidence of TCIPA is rather difficult to be capture in vivo. Thus, we divide the observation of TCIPA into three steps. In the first step, the initiation of TCIPA results in the depletion of platelets in the circulation to some degree, as shown in the peripheral platelet count test. Next, the TCIPA complex is formed during step two, which assists in tumor cell extravasation. The features of microthrombi containing a tumor cluster that we found in TCIPA in lung metastases by modified MSB correspond to the TCIPA complex described by MJ [44]. Finally, tumor cells colonize and fuse with platelets, leading to the expression of platelet-derived glycoproteins on the surface of tumor cells [47]. IHC results showed that CD42b and CD62p were significantly upregulated in TCIPA-induced lung metastases, suggesting that B16 cells carry platelets from the periphery to infiltrate lung metastases. Overall, we confirmed that our TCIPA model has indeed exhibits enhanced TCIPA.

Based on this TCIPA metastatic melanoma model, we focused on the mechanism through which TCIPA reshapes the TIME by inducing TAM recruitment and altering TAM polarization and PD-L1 expression. Increased TAM infiltration is associated with a high risk of recurrence and low survival in melanoma [48–50]. Among them, the M2-type-TAMs showed particularly increased infiltration in the TCIPA model lung metastases. The upregulation of PD-L1 on M2-type TAMs inhibits CD8^+^ T-cell toxicity [29, 51] and promotes the development of suppressive TIME. Moreover, lung metastasis decreased in the TCIPA model when an anti-PD-L1 antibody was administered, which further suggested that PD-L1 upregulation was partially mediated by TCIPA. Herein, we wonder what functions B16 cell-educated platelets performed independently. Unfortunately, the polarization and PD-L1 expression of B16 cell-educated platelets were superior to those of self-activated platelets but not those of the the equivalent B16 cells in vitro according to FCM. It is thus reasonable to speculate that the positive phenomena in vivo result from the recruitment of more TAMs by B16 cell-educated platelets, providing sufficient supply for subsequent reactions. Therefore, we focused on the mechanism by which TCIPA recruits more TAMs. As expected, we observed significant increases in the secretion of several cytokines, including CCL2, SDF-1, IL-1α, IL-1β, and the expression of their receptors CCR2, CXCR4 and IL-1R in the TCIPA model lungs. These pairs have been reported to have essential roles in the localization, mobilization and metabolism of TAMs [31, 32, 52], as well as in maintaining the immune-suppressive tendencies of the TIME [53] by promoting proliferation and differentiation of Th2 cells, protecting tumor cells from apoptosis and promoting angiogenesis.

One potential limitation of this study is that the findings were based on the use of a single melanoma cell line, B16F10, not multiple relevant cell lines, which may not be adequately robust. As we explored an immunology related mechanism underlying TCIPA-induced hematogenous metastasis, a complete immune system is the first consideration when establishing the model. Thus, we chose B16F10 cells in C57BL/6 mice to investigate the relationship among TCIPA, the TIME and metastasis. Whether this mechanism is applicable to other cell lines remains to be further verified in our future research.

## Conclusion

In conclusion, our study showed that TCIPA triggers TAM recruitment via the release of CCL2, SDF-1, IL-1α and IL-1β, which activate their receptors CCR2, CXCR4 and IL-1R, respectively, stimulating peripheral monocyte recruitment into tissues and differentiation into TAMs. Larger quantities of TAMs in the TCIPA model are polarized to the M2 type by B16 cell reprocessing, and their surface PD-L1 expression is upregulated, which ultimately helps B16 cells evade host immunity and accelerates melanoma hematogenous metastasis. Targeting B16 cell-educated platelets may provide a new therapeutic strategy for advanced melanoma patients with paraneoplastic thrombocytosis.

### Supplementary Information

**Additional file 1:** **Figure S1.** The ratio of PLT and B16 affects lung metastasis of TCIPA metastatic melanoma model (B16+PLT model). **Figure S2.** Tumor cells induced platelet aggregation by LTA assay. **Figure S3.** Effect of platelet on B16 cell proliferation in vitro. **Figure S4.** The stimulation of platelet on migration varies considerably in different tumor cell lines in vitro. **Figure S5.** The spleen and thymus index of TCIPA metastatic melanoma model (B16+PLT model). **Figure S6.** The immune cells altered by TCIPA in TIME.

**Additional file 2. **

## Data Availability

The datasets used and analyzed during the current study are available from the corresponding author [Guowang Yang and Ganlin Zhang] on reasonable request.

## Abbreviations

TCIPA: Tumor cell induced platelet aggregation
PD-L1: Programmed death ligant 1
TAM: Tumor associated macrophage
CCL2/MCP-1: C-c motif chemokine ligand 2/Macrophage chemoattractant protein-1
SDF-1: Stromal cell-derived factor
PD-1: Programmed cell death protein 1
ICIs: Immune checkpoint inhibitors
MM: Malignant melanoma
TIME: Tumor immune microenvironment
TEP: Tumor-educated platelet
PRP: Platelet-rich plasma
RBC: Red blood cell
WBC: White blood cell
LTA: Light transmission aggregometry
CCR2: C-C motif chemokine receptor 2
IL-1R1: Interleukin-1 receptor 1
CXCR4: C-X-C motif chemokine receptor 4
