# Supplementary material for: Tumor cell-induced platelet aggregation accelerates hematogenous metastasis of malignant melanoma by triggering macrophage recruitment
Source: J Exp Clin Cancer Res. 2023 Oct 23;42:277. doi: 10.1186/s13046-023-02856-1 (PMC10591353; doi:10.1186/s13046-023-02856-1)
Supplement: Supplementary file 1 — Additional file 1: Figure S1. The ratio of PLT and B16 affects lung metastasis of TCIPA metastatic melanoma model (B16+PLT model). Figure S2. Tumor cells induced platelet aggregation by LTA assay. Figure S3. Effect of platelet on B16 cell proliferation in vitro. Figure S4. The stimulation of platelet on migration varies considerably in different tumor cell lines in vitro. Figure S5. The spleen and thymus index of TCIPA metastatic melanoma model (B16+PLT model). Figure S6. The immune cells altered by TCIPA in TIME. [file 13046_2023_2856_MOESM1_ESM.docx]

**SUPPLEMENTARY MATERIAL 1**

**Tumor Cell-Induced Platelet Aggregation (TCIPA) Accelerates Hematogenous Metastasis of Melanoma by Triggering** **Macrophages Recruitment**


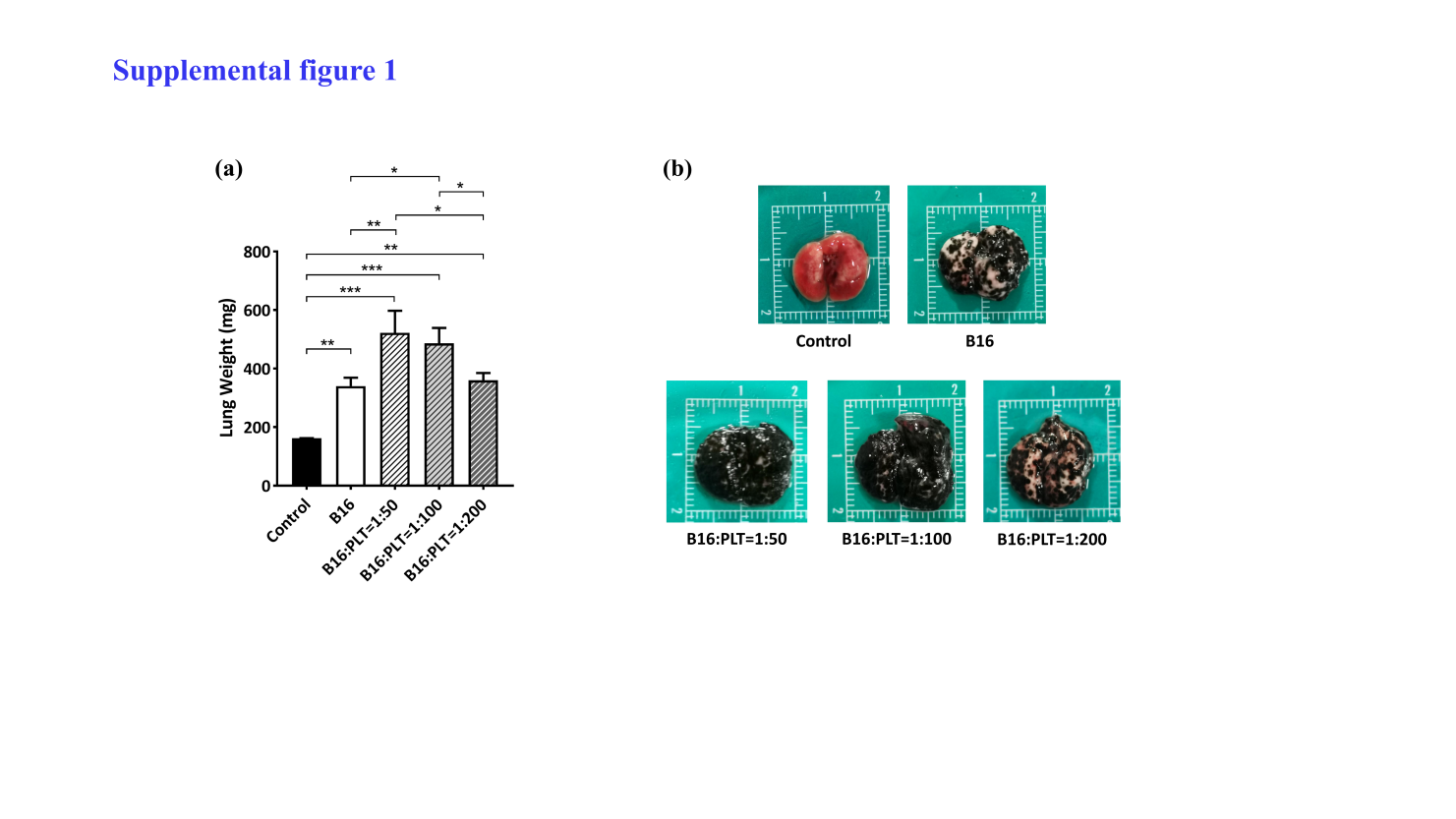


**Figure S1. The ratio of PLT and B16 affects lung metastasis of TCIPA metastatic melanoma model (B16+PLT model).** (a) graph for lung weight of B16:PLT=1:50 model, B16:PLT=1:100 model, B16:PLT=1:200 model, B16 model and control groups. **p*≤0.05, ***p*≤0.01, ****p*≤0.001, N=5. (b) Photos of lung metastases.


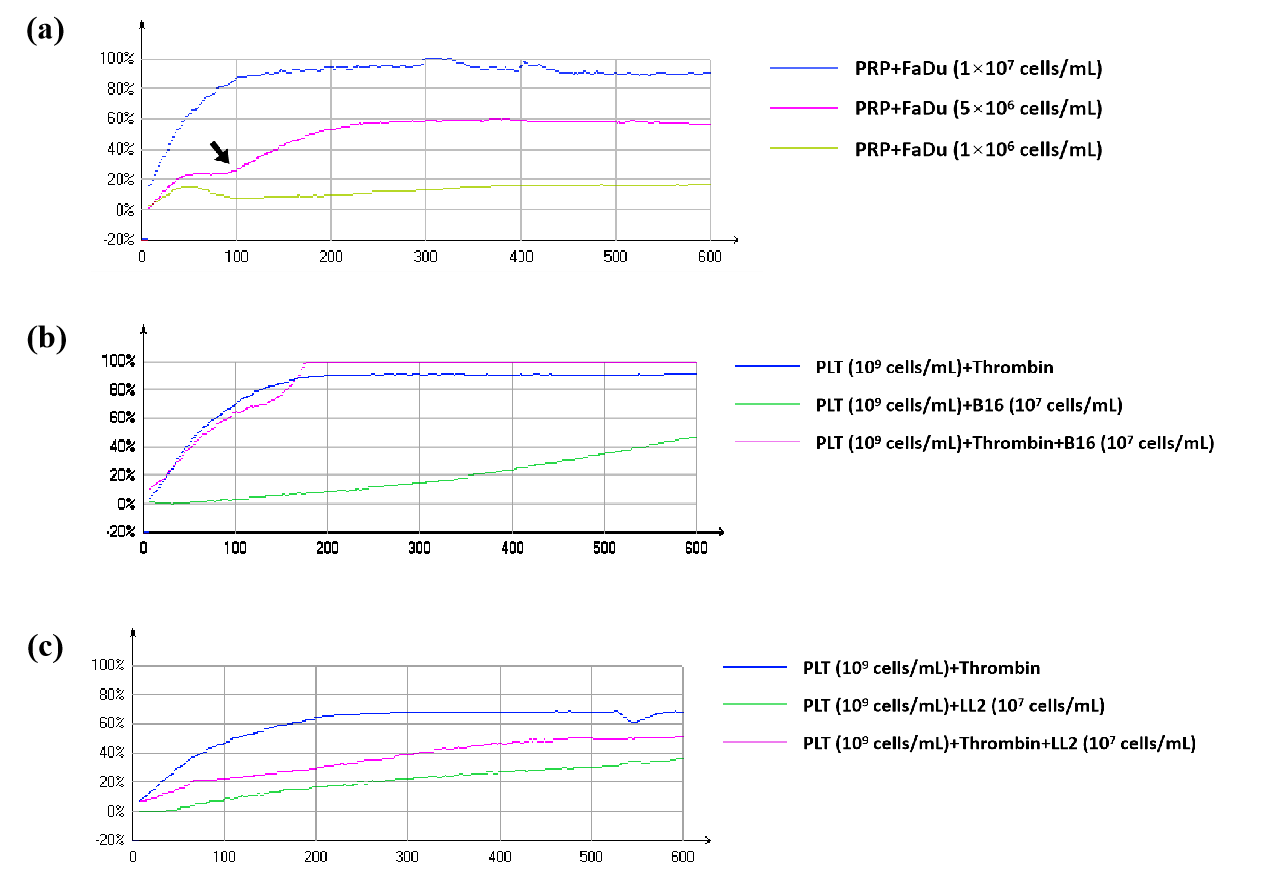


**Figure S2. Tumor cells induced platelet aggregation by LTA assay.** (a) FaDu cells in different concentrations induced platelet aggregation. FaDu cells at a final concentration of 1×10^7^ cells/mL was superior to 5×10^6^ cells/mL and 1×10^6^ cells/mL in their ability to induce platelet aggregation. (b and c) Both B16 and LL/2 cell at a final concentration of 1×10^7^ cells/mL were able to induce platelet aggregation, and thrombin was used as a positive control.


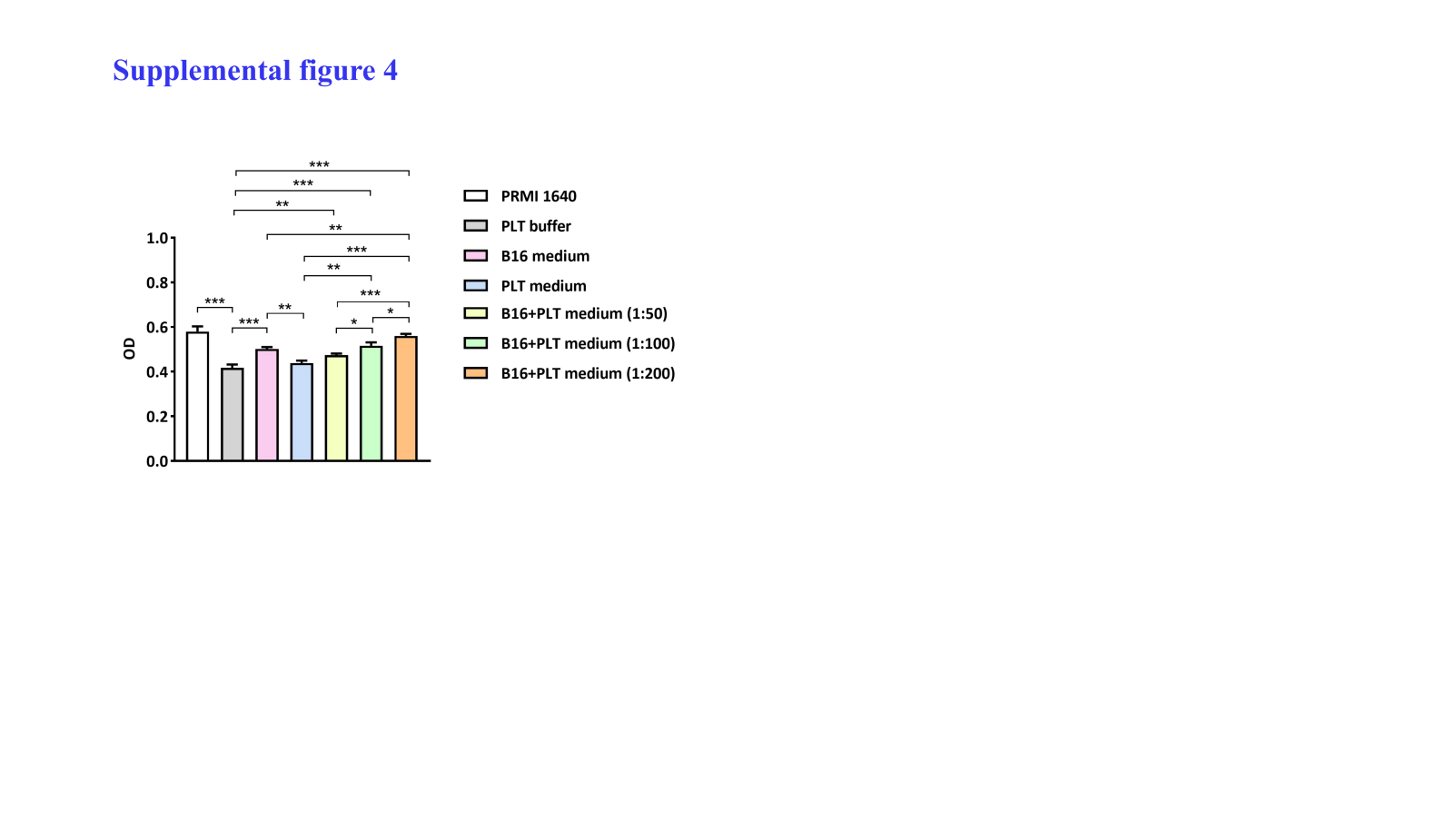


**Figure S3. Effect of platelet on B16 cell proliferation in vitro.** **p*≤0.05, ***p*≤0.01, ****p*≤0.001, N=6.


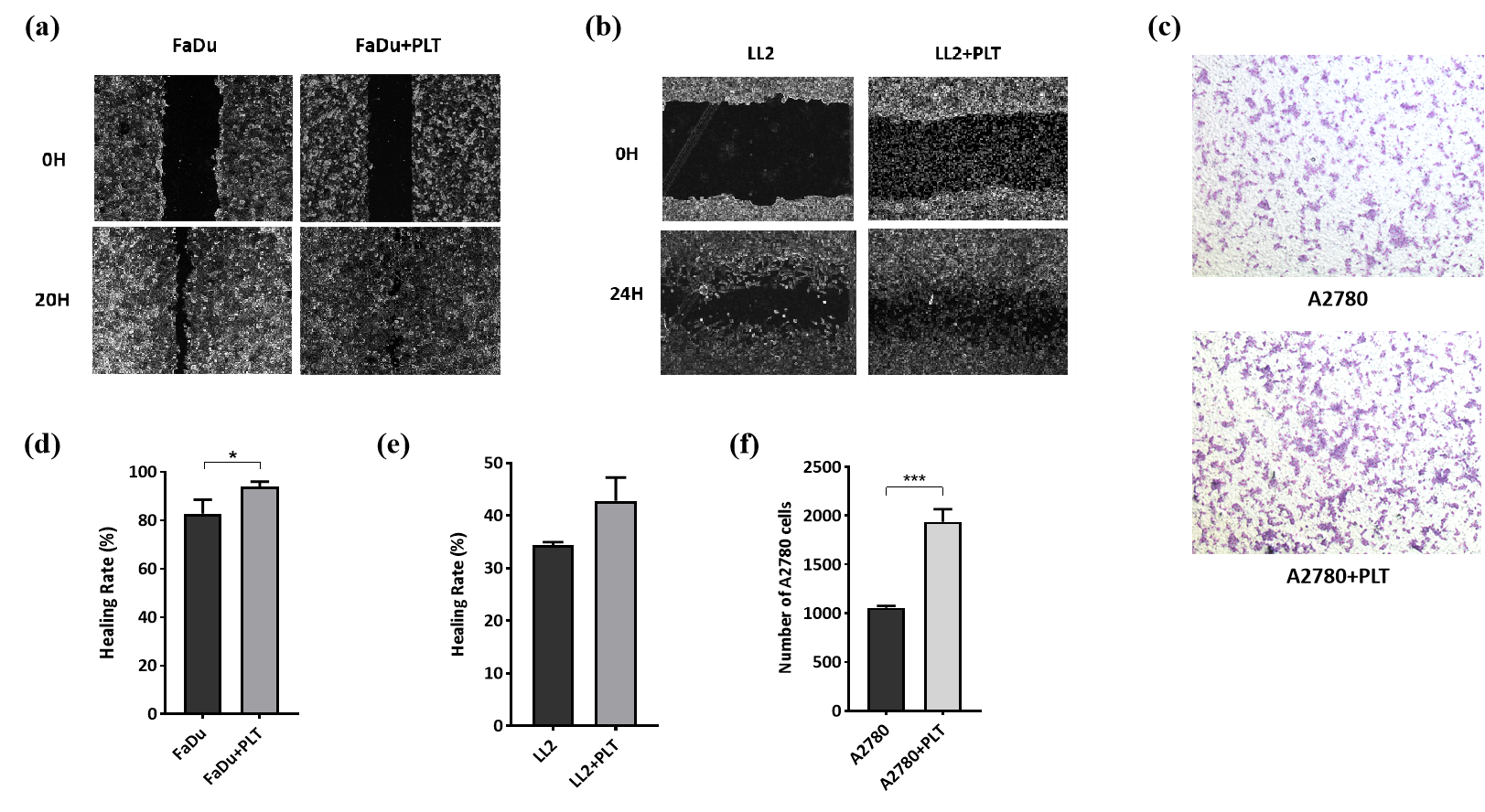


**Figure S4.** **The stimulation of platelet on migration varies considerably in different tumor cell lines in vitro.** The migratory ability of platelet-educated FaDu cell is found to be enhanced compared to FaDu cell itself. (a and d, **p*≤0.05, N=3). The phenomenon is also verified in LL/2 (b and e, N=3) ,and A2780 cell lines (c and f, ****p*≤0.001, N=5).


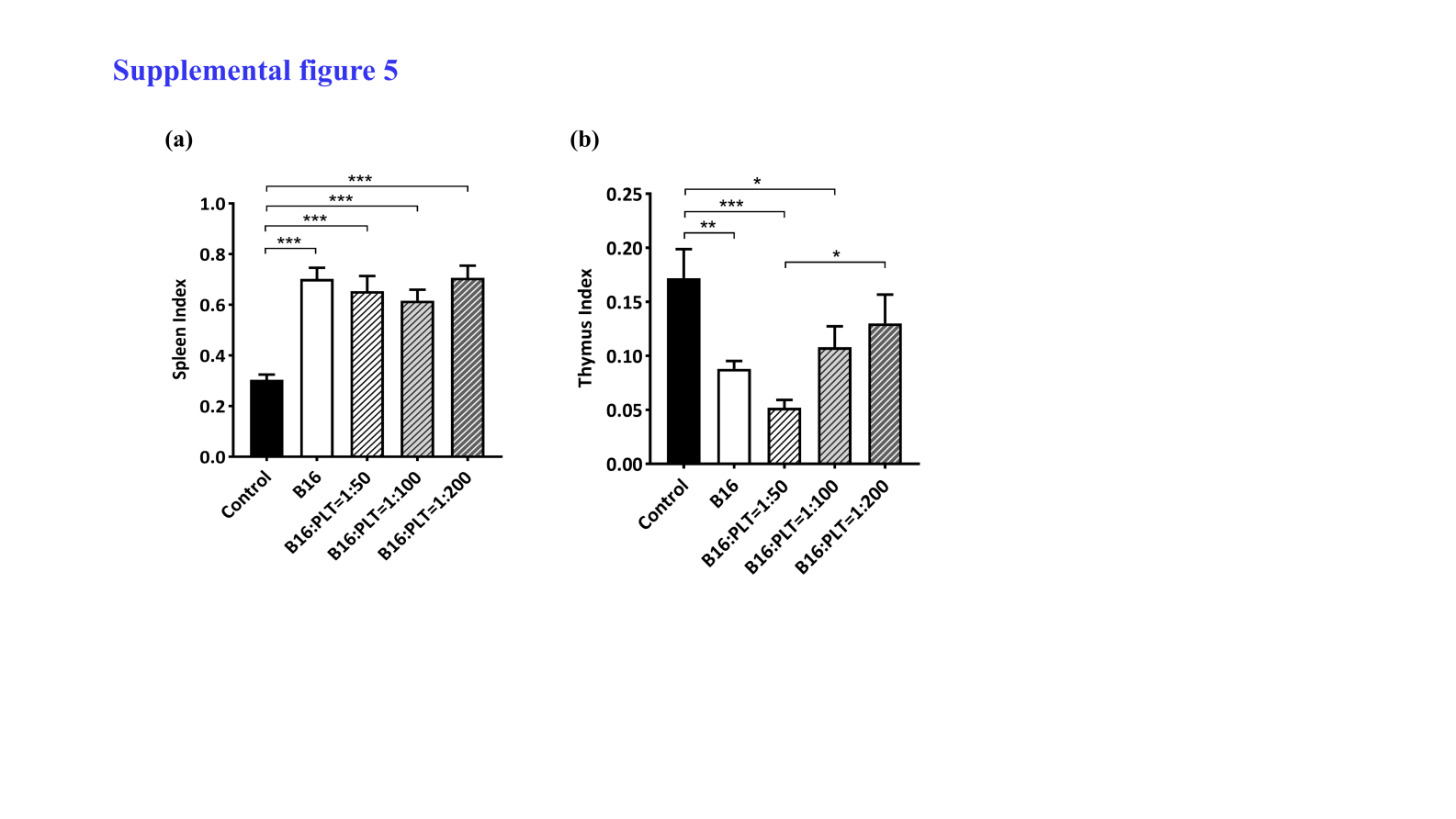


**Figure S5. The spleen and thymus index of TCIPA metastatic melanoma model (B16+PLT model).** (a) Spleen index (spleen weight/body weight×100). (b) Thymus index (thymus weight/body weight×100). **p*≤0.05, ***p*≤0.01, ****p*≤0.001, N=5.


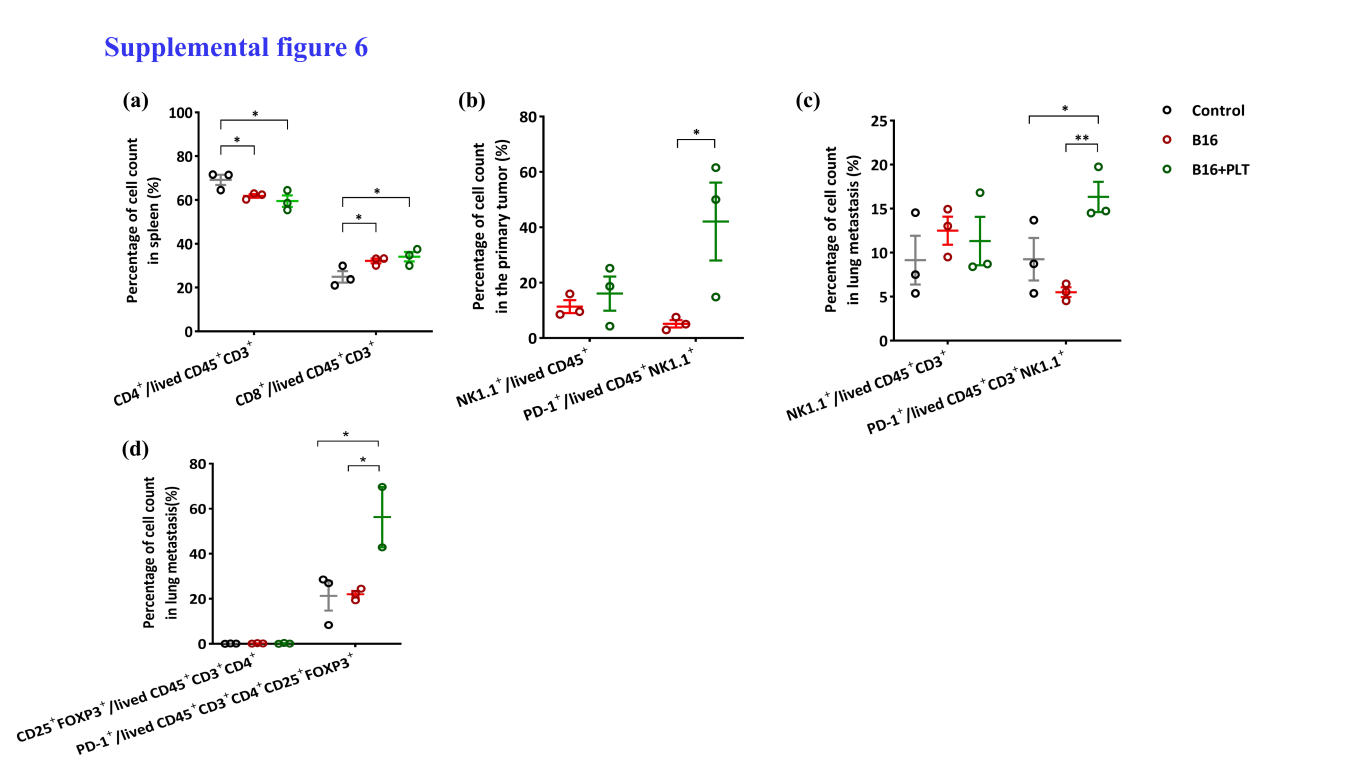


**Figure S6. The immune cells altered by TCIPA in TIME.** (a) The percentage of CD4^+^ and CD8^+^ T cells in spleen. (b) The percentage of NK cells in the primary tumor and their PD-1 expression. (c) The percentage of NKT cells and their PD-1 expression in lung metastases. (d) The percentage of Treg cells and their PD-1 expression in lung metastases. **p*≤0.05, ***p*≤0.01, N=3.
