# Supplementary material for: Tumor cell-induced platelet aggregation accelerates hematogenous metastasis of malignant melanoma by triggering macrophage recruitment
Source: J Exp Clin Cancer Res. 2023 Oct 23;42:277. doi: 10.1186/s13046-023-02856-1 (PMC10591353; doi:10.1186/s13046-023-02856-1)
Supplement: Supplementary file 2 — Additional file 2. [file 13046_2023_2856_MOESM2_ESM.doc]

**SUPPLEMENTARY MATERIAL 2**

1. Dry sections in oven 60°C for at least 30 minutes.
2. Xylene Ⅰ --5 minutes
3. Xylene Ⅱ --5 minutes
4. Xylene Ⅲ --5 minutes
5. 100% alcohol Ⅰ --3 minutes
6. 100% alcohol Ⅱ --3 minutes
7. 95% alcohol --1 minute
8. 70% alcohol --1 minute
9. 50% alcohol --1 minute
10. Wash slides in running tap water --5 minutes
11. Place into hypo solution (sodium thiosulphate, hyposulfite) --4min, room temperature (RT)
12. Wash slides in PBS --30 seconds
13. Place into alum-celestine blue solution --4min, RT
14. Wash slides in PBS --30 seconds
15. Place into Mayer’s hematoxylin --4min, 4°C, dark place
16. Place into ethanol hydrochloride --5 seconds
17. Wash slides in running tap water --10 minutes
18. Rinse with 95% alcohol --30 seconds
19. Place in Martius yellow solution --3 minutes
20. Wash slides in PBS --30 seconds
21. Place in crystal scarlet --10 minutes, RT
22. Wash slides in PBS --30 seconds
23. Differentiate with phosphotungstic acid until the red color of collagen disappears --5~10 minutes, RT, dark place
24. Rinse with distilled water --30 seconds
25. Place in methyl blue until collagen is blue --observed every 2 minutes for up to 10 minutes
26. Rinse with 1% aqueous acetic acid --1 minute
27. 5% alcohol --5 seconds
28. 50% alcohol --5 seconds
29. 70% alcohol --5 seconds
30. 90% alcohol --5 seconds
31. 95% alcohol Ⅰ--5 seconds
32. 95% alcohol Ⅱ--5 seconds
33. 100% alcohol Ⅰ--10 seconds
34. 100% alcohol Ⅱ--10 seconds
35. 100% alcohol Ⅲ --10 seconds
36. Xylene Ⅰ --1 minute
37. Xylene Ⅱ --1 minute
38. Xylene Ⅲ --1 minute
39. Xylene Ⅳ --1 minute
40. Seal with neutral balsam
